# Disparity and Diversity in NSCLC Imaging and Genomics: Evaluation of a Mature, Multicenter Database

**DOI:** 10.3390/cancers15072096

**Published:** 2023-03-31

**Authors:** Andres Kohan, Roshini Kulanthaivelu, Ricarda Hinzpeter, Zhihui Amy Liu, Claudia Ortega, Natasha Leighl, Ur Metser, Patrick Veit-Haibach

**Affiliations:** 1 Joint Department of Medical Imaging, University Health Network, Mount Sinai Hospital and Women’s College Hospital, University of Toronto, Toronto, ON M5G 1X6, Canada; 2 Department of Biostatistics, Princess Margaret Cancer Centre, University Health Network, Dalla Lana School of Public Health, University of Toronto, Toronto, ON M5G 2C4, Canada; 3 Thoracic Medical Oncology Group, Princess Margaret Cancer Centre, University Health Network, Toronto, ON M5G 2C4, Canada

**Keywords:** NSCLC, lung cancer, genomics, disparity, diversity, imaging

## Abstract

**Simple Summary:**

Lung cancer remains the leading cancer-related death across North America. As imaging is fundamental for its diagnosis, treatment, and follow-up, the presence of healthcare disparities at this level needs to be identified. Using a multicenter collated database, we aimed to identify the existence of such disparities while describing the composition and behavior of our study population. Our results highlight the relevance of adequate access to imaging while being treated for NSCLC that could help pivot treatments early in both clinical and experimental settings.

**Abstract:**

Lung cancer remains the leading cancer-related death across North America. Imaging is fundamental. Recently, healthcare disparities came into research focus. Our aim was to explore disparity from an imaging, genetic, and outcome perspective. We utilized the AACR Project GENIE Biopharma Consortium (BPC) dataset v 1.1 to build a collated NSCLC dataset. Descriptive and analytical statistics were applied according to data characteristics. From 1849 patients, mean age was 64.4 y (±10.5), 58% (*n* = 1065) were female, 23% (*n* = 419) never smoked, 84% (*n* = 1545) were of white race, and 57% (*n* = 1052) were < stage III. No difference (*p* > 0.05) was found for baseline imaging by race. White race showed higher 3-month surveillance imaging (*p* = 0.048) and a baseline stage < IV (OR 0.61). KRAS (33.3 vs. 17.9%), STK11 (14.8 vs. 7.3%), and KEAP1 (13.3 vs. 5.3%) mutations were predominant among white patients while EGFR mutation (19.2 vs. 44.1%) was less predominant. Mutations in TP53 or KEAP1 had worse PFS and OS. The latter was also reduced in STK11, KRAS + STK11, and KRAS + KEAP1 mutations. Meanwhile, EGFR mutation had increased OS. Multivariate analysis showed that progression on imaging at 3 or 6 months (HR 1.69 and 1.43, respectively), TP53 (HR 1.37) and KRAS (HR 1.26) had lower OS while EGFR and LRP1B (HR 0.69 and 0.39, respectively) had higher OS. No racial disparity at baseline imaging was observed. Higher initial stages among non-white patients might reflect inequalities in accessing healthcare. However, race wasn’t associated to OS. Finally, progression in imaging at 3 or 6 months showed the higher hazard ratios for death.

## 1. Introduction

Lung cancer remains the leading cause of cancer death across North America [1], affecting up to 29,800 Canadians [2] and 235, 760 Americans [3] each year. Non-small cell lung cancer (NSCLC) accounts for 84% of all lung cancer diagnoses [2] and poses a significant burden to the individual as well as the healthcare system [4,5].

Disparities in cancer outcomes have been partly described previously, particularly among patients of different race/ethnicities [6,7]. The causes of healthcare disparity can be broadly separated in social (structural, socioeconomic, socio-environmental, and behavioral), and biological (genomic profiles that determine tumor biology, aggressiveness, and response to treatment) [8,9]. In this context, imaging plays a fundamental role among patients with NSCLC, from initial diagnosis to surveillance and restaging, and conduction of imaging is also partly influenced by the mentioned disparities.

Current guidelines recommend that patients with NSCLC are evaluated with CT, PET/CT +/− MRI Brain for the purposes of staging [10,11], and that following curative therapy for patients with stage I-III disease, patients undergo surveillance CT every 6 months for 2 years [12,13]. In light of these existing guidelines for NSCLC imaging, there should, in theory, be only minor discrepancy between the types and frequency of imaging studies utilized among patients with NSCLC. A study from 2011, however, demonstrated that Hispanic and non-white patients were less likely to receive PET/CT imaging during staging for NSCLC [14]; a finding that has been confirmed in a study from Colorado [15]. This is relevant because PET/CT has been shown to result in improved staging corresponding to a 20% improvement in 1-year survival for NSCLC [16]. There are, however, currently no studies available evaluating potential disparities among imaging studies employed at both diagnosis and surveillance.

Previous studies have shown that even when factors contributing to socioeconomic healthcare disparity are accounted for, a difference in cancer outcomes among different racial and ethnic groups remains, suggesting an intrinsic difference in tumor biology and treatment response [8,9]. For example, disparities in prostate and breast cancer survival remain among black patients even after controlling for socioeconomic factors, with more aggressive forms of prostate cancer seen among black patients [9].

In NSCLC, genomic profiling has already successfully identified epidermal growth factor receptor (EGFR)-activating somatic mutations among Asian patients [17,18] with the subsequent effective use of targeted therapy. Research so far has mostly focused on genomic profiling of NSCLC in Asian or African American/Black populations [18,19,20]. There is, however, a relative paucity of studies looking at the relationship between genomic markers and other racial/ethnic groups, and there have been no studies looking specifically at imaging-derived response and its relationship with different genomic markers and racial/ethnic groups. Such relations, if identified, could potentially facilitate and accelerate the development of further targeted therapies and help reduce the disparity between different racial/ethnic groups.

The aim of our study was, therefore, to examine disparities in patients with NSCLC from an imaging perspective and explore its relationship with genomic and racial diversity.

## 2. Materials and Methods

AACR Project GENIE is a publicly accessible cancer registry of real-world data assembled through data sharing between 19 leading international cancer centers. The AACR Project GENIE Biopharma Consortium (BPC) dataset v 1.1 is a dataset of curated clinical, imaging, and genomic data of patients with NSCLC from four different academic institutions across North America (Memorial Sloan Kettering Cancer Center (MSKCC), Manhattan, NY, USA, Dana Farber Cancer Institute (DFCI), Chestnut Hill, MA, USA, Vanderbilt Ingram Cancer Center (VICC), Nashville, TN, USA, and Princess Margaret Cancer Centre (PMH), Toronto, ON, Canada [21]. The Pathology, Radiology, Imaging, Signs, Symptoms, BioMarker, Medical Oncology Notes (PRISSMM™) data model was used to develop the NSCLC v1.1 dataset. Inclusion criteria for the dataset included patients aged 18 and above with NSCLC Stage I-IV at diagnosis with a genomic sequencing report from MSKCC, DFCI, VICC, or PMH between 1 January 2014, and 31 December 2017 and a minimum of two years of follow-up after sequencing.

Race was categorized as White, Black, American Indian, Aleutian or Eskimo, Chinese, Japanese, Filipino, Hawaiian, Korean, Vietnamese, Laotian, Hmong, Kampuchean, Thai, Asian Indian or Pakistani NOS, Asian Indian, Pakistani, Micronesian NOS, Chamorro/Chamoru, Guamanian NOS, Polynesian NOS, Tahitian, Samoan, Tongan, Melanesian NOS, Fiji Islander, New Guinea, Other Asian, Pacific Islander NOS, Other, or Unknown.

The number of imaging reports (CT, MRI, and PET/CT scans) starting in the month/year of inclusion in the BPC project cancer diagnosis was collated. Curated imaging report assessment (presence/absence of cancer) and change in cancer status (improving/responding, stable/no change, mixed, progressing/worsening/enlarging) were also collated from the database. The curated imaging data is obtained from RECIST-based imaging reports used in everyday clinical practice. For the purposes of assessing initial staging, the number of imaging studies performed after the initiation of chemotherapy were assessed. Meanwhile, for surveillance imaging, the number of imaging studies performed after diagnosis or the initiation of therapy, if performed, were assessed. Complete baseline imaging was defined as having a chest CT, a brain MRI, and a whole-body PET/CT prior to start of treatment. The presence of complete baseline imaging (CT + MRI + PET/CT) and the total number of individual imaging studies (CT, MRI, or PET/CT) at baseline and during follow-up were compared by race (white vs. non-white).

The 10 commonest genomic mutations (frequency ≥ 10%), were selected for analysis within the study (TP53, KRAS, LRP1B, EGFR, STK11, KEAP1, PRKDC, RBM10, KMT2D, and GRM 3). Imaging response was assessed in a subgroup of patients post chemotherapy at 3, 6, and 12 months.

Progression-free survival (PFS) was defined as time from diagnosis or time from start of initial chemotherapy, if recorded, to first mention of ‘progression’ on imaging. Overall survival (OS) was defined as time from diagnosis or commencement of initial treatment, if recorded, to death/last follow-up. OS and PFS outcomes were assessed and compared between races and for different genetic markers.

### Statistical Analysis

Patient characteristics were summarized using mean and standard deviation or median and inter-quartile range for continuous variables. Frequency and proportions were used for categorical variables. For our analysis, the race of patients was dichotomized as white or non-white. Reason for the dichotomization was that, despite having granular information about multiple different ethnicities, the non-white groups were very diverse and, therefore, too small for robust statistical analysis in this context. Characteristics were compared between race groups using Wilcoxon’s rank sum test and Chi-squared or Fisher’s exact test.

Comparisons between the dichotomized race groups regarding all available imaging procedures were evaluated at baseline, 3 months, 6 months, and 1 year. The evaluation of distribution of each genetic marker was conducted using Chi-squared or Fisher’s exact test depending on expected cell value.

Logrank test was used to compare OS and PFS between the groups evaluated in this analysis, with Kaplan-Meier curves drawn for additional illustration, as shown below. A multivariable Cox proportional hazards model was fitted to assess the association between progression observed in imaging at different (as defined above) timepoints, the available races, and genetic markers with OS or race as well as genetic markers with PFS. All analyses were run using R v4.0.2. Results for all the above-mentioned evaluations were considered statistically significant if *p* < 0.05.

## 3. Results

### 3.1. Patient Characteristics

Out of the 1849 patients analyzed, 58% (*n* = 1065) were females, 23% (*n* = 419) had never smoked, and 84% (*n* = 1545) were classified as of the white race. Mean (SD) age of the cohort was 64.4 y (±10.5) and the majority presented with stage III or lower (57%, *n* = 1052). Population characteristics are summarized in Table 1.

Regarding their initial stage at moment of diagnosis, non-white patients had significantly higher stages than white patients (*p* = 0.004). Furthermore, white patients had an OR of 0.61 (95% CI 0.46–0.81, *p* = 0.0005) of having an initial stage IV and an OR of 1.67 (95% CI 1.17–2.40, *p* = 0.004) for an initial stage I when compared to non-white patients.

### 3.2. Imaging Studies and Race

When evaluating completeness of baseline imaging by race (Table 2), no significant difference was found (*p* = 0.4). There was also no statistically significant difference found between white vs. non-white patients when evaluating single-imaging modalities at baseline (i.e., CT, MRI, and PET/CT) (Table 2). Thus, all patients received similar imaging-based diagnosis at baseline with all evaluated imaging modalities. However, there was a tendency for white patients to undergo more PET/CT compared with non-whites (70% for white vs. 64% for non-white, *p* = 0.077) at baseline.

When analyzing surveillance imaging performed at 3, 6 and 12 months after diagnosis or commencement of chemotherapy, if recorded, we found that white patients had a statistically higher rate of surveillance imaging at the 3-month follow-up time point (68 vs. 61%, *p* = 0.048).

### 3.3. Imaging-Derived Response and Genomic & Racial Diversity

We compared the distribution of the 10 most encountered genetic mutations among patients with NSCLC (Table 3) between white and non-white patients. White patients had statistically significant more KRAS (33.3% vs. 17.9%), STK11 (14.8% vs. 7.3%), and KEAP1 (13.3% vs. 5.3%) mutations and non-white patients had significantly more EGFR mutations (44.1% vs. 19.2%).

Overall, no statistically significant differences were found on imaging concerning PFS between the analyzed groups (white-vs. non-white patients). When analyzing imaging outcomes at the three different imaging follow-up time points individually, there was no difference at the 3- and 6-month follow-up time point between the groups. Meanwhile at the 1-year follow-up time point, non-white patients had significantly different imaging findings (*p* = 0.0026). However, the hazard ratios for progression as reported by imaging based on race were not significant at any follow-up time point. Thus, the difference seen at 1-year follow-up might be related to other outcomes (stable disease, complete response, etc.).

When performing univariate analysis of imaging-detected progression at the individual genetic mutation level, patients with a TP53 mutation showed worse PFS (*p* = 0.006). This was also true for patients presenting the mutation KEAP1 (*p* = 0.03). Meanwhile, in the chemotherapy subgroup that had the EGFR mutation, better PFS (*p* = 0.0015) was observed when compared to their counterparts.

**Figure 1:**
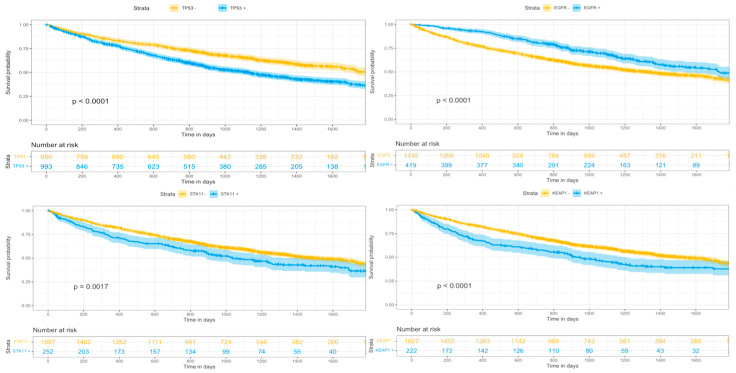
Overall survival curves for significant genetic markers in all patients.

**Figure 2:**
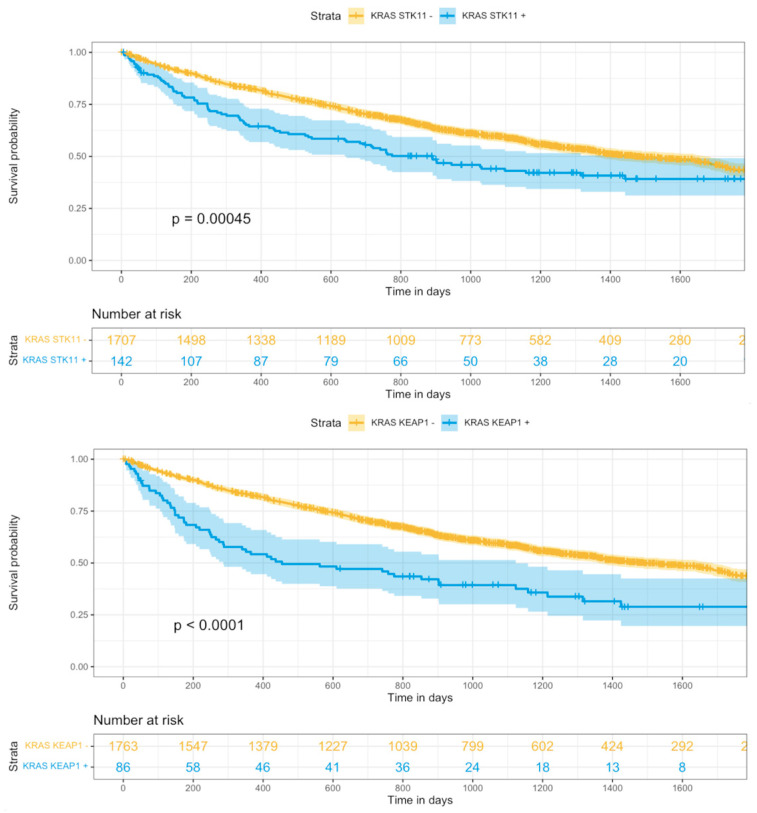
Overall survival curves for KRAS + STK11 and KRAS + KEAP1 markers in all patients.

Regarding OS, patients with TP53, STK11, and KEAP1 mutation showed a significantly worse OS compared to those patients without those mutations (Figure 1). Furthermore, combined mutation of KRAS + STK11 and KRAS + KEAP1 was also associated with significantly worse OS (Figure 2). This was also true for the subgroup of patients that underwent chemotherapy. On the contrary, for the entire patient population (Figure 1) including those that received chemotherapy, having an EGFR mutation meant a significantly improved OS.

When correlating OS and race within the entire database, there was no statistically significant difference between white and non-white patients (*p* = 0.057).

The Cox proportional-hazards model analysis done on the genetic markers, image derived progression at 3, 6 or 12 months, and race showed that the variable that showed the highest hazard ratio for OS was image-derived progression at 3 months (Table 4). Meanwhile, progression at 6 months and positive TP53 or KRAS were also associated with an increased risk for death, while positive EGFR or LRP1B were associated with a decreased risk for death. The same findings were observed when the analysis was repeated in the chemotherapy group.

## 4. Discussion

Our study investigates both disparity in imaging between different racial groups obtained at baseline and surveillance as well as genomic diversity in NSCLC among different racial groups, and their relationships with image-derived response. Compared to previous literature indicating a disparity in imaging studies obtained among patients of different race, our study only showed disparity at the 3-month surveillance time point. When exploring genomic diversity among our population, white patients had statistically significant higher mutations of KRAS, STK11, and KEAP1. Meanwhile, EGFR was more frequently mutated among non-white patients. TP53 and KEAP1 were associated with decreased PFS in overall population and EGFR with better PFS in the chemotherapy group. Finally, when controlling for multiple variables image-derived progression evidenced at 3 months, in particular, and at 6 months were the most relevant in OS prognostication both in the general sample and the chemotherapy subgroup.

### 4.1. Disparity in Imaging Studies and Race

Despite evidence in the literature indicating a disparity in imaging obtained among patients of different race [8,15,22,23] and the impact this disparity has on their mortality [24,25], our study does not demonstrate a statistically significant difference in imaging obtained at baseline. This may be, in part, due to the relatively low percentage of non-white patients within the AACR database (13%, *n*= 245).

There was no statistical difference in complete baseline imaging when comparing white vs. non-white patients. However, the high percentage of patients with incomplete imaging assessment at baseline (white 56% and non-white 60%) struck us as odd despite endorsement from current clinical guidelines [10,11,13].

When all the components of baseline imaging (CT, MRI, and PET/CT) were compared separately, the lack of significance remained unaltered. Nevertheless, there seemed to be a tendency towards racial disparity in PET/CT (70% white vs. 64% non-white, *p* = 0.0768), a finding confirmed in other studies [14,15]. It is noteworthy that higher rates of PET/CT and MRI were performed at baseline in both groups (Table 2) and in the overall population when compared to diagnostic chest CT alone (69% vs. 68% vs. 43%, respectively). This is somewhat unusual, since chest CT is among the first imaging modalities used for patients presenting with symptoms. Furthermore, CT performed for other reasons, alongside chest x-ray, is an important source of incidentally found pulmonary lesions, which subsequently undergo work-up for lung nodules/masses [26]. Also, low-dose CT has been recommended for high-risk patients from as early as 2011 [27]. Therefore, we hypothesize that this difference might be related to lack of reporting of a baseline imaging CT within the database.

In the context of surveillance imaging, we observed a higher rate of follow-up imaging performed at 3 months among white patients compared to non-white (68% white vs. 61% non-white) while no difference was observed at 6- and 12-month follow-up. Meanwhile Kunitomo et al. [28] found in a meta-analysis that there was lower adherence to lung cancer screening follow-up in patients of black race compared to patients of white race. This discrepancy could be related to the fact that our study cohort consists of patients from multiple tertiary cancer centers receiving specialist treatment. In that sense, we found no evidence of disparity once inside the system but in the difference at 3-month follow-up time point. However, the difference we found in staging between groups at diagnosis confirms Kunitomo et al. [28] findings and could be in line with what has been reported by DeSantis et al. [29], potentially indicating that later stages at diagnosis were due to socioeconomic barriers blocking timely access to high-quality medical care. However, we have no information on the socioeconomic status of the patients within the database to confirm the latter.

Despite the higher stage at initial diagnosis for non-white patients, we did not find a difference in OS between groups. Furthermore, at a *p* = 0.057 our population shows a tendency for higher OS for non-white patients, something that has been reported differently in other papers [1,30]. This could also explain why a higher total number of imaging reports were observed in the non-white group (22.4 vs. 18.6, *p* < 0.001), as patients who live longer tend to have more studies. A possible reason behind these findings is likely related to the genetic characteristics of tumors within those groups.

### 4.2. Genomics, Racial Diversity, and Image Derived Response

TP53, KRAS, EGFR, STK11, and KEAP1 were the most encountered genetic mutations in our patient population. The top three driver genes in the literature among patients with NSCLC are EGFR, ERBB1, and KRAS [31,32]. White patients had statistically significant more KRAS, STK11, and KEAP1 mutations while higher rates of EGFR mutation was seen among non-white patients. These findings correlate with those previously seen in the literature, where KRAS and EGFR are often seen as mutually exclusive mutations with higher rates of KRAS in Western countries and EGFR in Asiatic ones [20,31,33,34].

Interestingly, in contradiction to the published literature, we observed no difference in OS between groups [1,30]. Furthermore, despite presenting with higher stage, non-white patients showed a possible tendency for improved OS (*p* = 0.057). In opposition to previous studies, when controlling for multiple variables, only progression evidenced by imaging at 3 and 6 months and the genetic profiles KRAS, TP53, EGFR, and LRP1B were identified as significantly affecting OS. Furthermore, one of our most relevant findings is that imaging-based progression at different time points (3 and 6 months) has the highest OR for death in overall population and the chemotherapy subgroup (Table 4) in the multivariable analysis. This aligns with what Kehl et al. [35] found where radiologic determination of PFS was one of the best correlated surrogate endpoints with OS.

In the current era of precision medicine, genetic profiling and targeted therapies are a desirable resource [36]. However, our study shows that early identification of relapse or progression by imaging is the main prognosticator regarding OS. This is specifically important in several ways. On one hand, this is one of the first studies confirming a statistically significant role of imaging at specific time points for NSCLC patients based on mature registry data. Additionally, it provides imaging specialists and treating physicians with an additional and reliable tool for therapy adjustments, and places additional emphasis on the importance of the imaging findings. This additional prognostication resource provides the treating physician with a tool to add further precision concerning therapy decision, i.e., for early intensification or maybe also change of the therapeutic goals. However, while several therapies are very imaging-dependent these days, i.e., several types of lymphoma therapies are heavily PET/CT driven, this is not a universal finding [37,38].

### 4.3. Limitations

This study has several weaknesses, most of them associated to the use of a secondary source of information. The most relevant one, given our attempt to address disparities, is the disproportionate distribution of racial characteristics which may bias our results towards white population. Another weakness of our study is that we could only evaluate baseline imaging on patients that had chemotherapy. It was the only sign of treatment available in the database that we could use to establish what was done as baseline imaging. Unfortunately, surgical treatment and its data were not detailed in this iteration of the database. Finally, we worked with curated imaging reports and not the actual DICOM images.

## 5. Conclusions

Our study is one of the first exploring disparity in imaging between different racial groups obtained at both baseline and surveillance as well as correlating genomic diversity in NSCLC and its relationship with image derived response. No definite racial disparity in number of performed imaging procedures at baseline was identified, but we did identify a disparity in overall surveillance imaging at 3 months. Our finding that non-white patients had a higher stage at diagnosis is possibly related to inequalities in access to healthcare, rather than inequalities within the healthcare system itself. Finally, imaging-based progression within the first year presents the highest risk for death for all patients regardless of race or genetic markers.

## Figures and Tables

**Table 1:** Population characteristics.

	*n* = 1849
Sex (n)	
Male	42% (784)
Female	58% (1065)
Race (n)	
White	83.6% (1545)
Black	5.1% (94)
Chinese	4.7% (87)
Other Asian	1.8% (33)
American Indian, Aleutian or Eskimo	0.2% (4)
Hawaiian	0.1% (2)
Other	1.4% (25)
Unknown	3.2% (59)
Age at Dx (Sd)	64.4 (10.5)
NSCLC Type (n)	
Adenocarcinoma	68% (1262)
Squamous Cell	9% (170)
Other	23% (417)
Stage at Dx (n) *	
I-III	57% (1052)
IV	43% (793)
Smoking History (n)	
Current	14% (259)
Former <1 y	12% (218)
Former >1 y	51% (950)
Never	23% (419)
Other Cancers (n)	29% (540)
Cancer-Related Death (n)	49% (912)
Progression (n)	
2–4 mo	10% (179)
5–7 mo	10% (187)
10–14 mo	17% (311)
Within the 1st year	31% (572)
Chemotherapy (n)	65% (1197)
Platinum Based Therapy (n)	69% (828)
Complete Baseline Imaging (n)	43% (516)
Progression (n)	
2–4 mo	12% (143)
5–7 mo	13% (152)
10–14 mo	22% (268)

* There were four missing values.

**Table 2:** Baseline assessment of patients.

Race (*n* = 1155 *)	Baseline Imaging		Baseline CT ^†^		Baseline MR		Baseline PET	
	+	−	+	−	+	−	+	−
White	421 (44%)	540 (56%)	421 (44%)	540 (56%)	667 (69%)	294 (31%)	676 (70%)	285 (30%)
Non-White	78 (40%)	116 (60%)	78 (40%)	116 (60%)	123 (63%)	71 (36%)	124 (64%)	70 (36%)

* Unknown Race was excluded from analysis—^†^ All patients that had baseline CT had Baseline MRI and PET.

**Table 3:** Genetic marker distribution globally and by race.

Genetic Markers (N)	All (N = 1849)	White (N = 1545) *	Non-White (N = 245) *	*p*
TP53	53.7% (993)	54.1% (836)	50.6% (124)	0.11
KRAS	30.8% (569)	33.3% (514)	17.9% (44)	**0.02**
EGFR	22.7% (419)	19.2% (296)	44.1% (108)	**<0.01**
STK11	13.6% (252)	14.8% (229)	7.3% (18)	**0.01**
KEAP1	12.0% (222)	13.3% (206)	5.3% (13)	**<0.01**
KMT2D	9.3% (172)	9.9% (153)	6.1% (15)	0.06
RBM10	6.9% (127)	7.2% (112)	5.3% (13)	0.23
PRKDC	5.0% (92)	5.4% (83)	3.3% (8)	0.53
LRP1B	1.0% (18)	0.9% (15)	0.8% (2)	0.89
GRM3	0.3% (6)	0.3% (4)	0.8% (2)	0.85
KRAS + STK11^+^	7.5% (140)	8.5% (132)	3.2% (8)	**<0.01**
KRAS + KEAP1^+^	4.5% (85)	5.4% (84)	0.4% (1)	**<0.01**

* Unknown Race (*n* = 59) was excluded from analysis.

**Table 4:** Cox regression for OS.

Predictors	All Patients			Chemotherapy Group		
	Death			Death		
	Hazard Ratio	95% CI	*p*	Hazard Ratio	95% CI	*p*
Progression at 3 mo	1.70	1.40–2.05	**<0.01**	1.92	1.55–2.38	**<0.01**
Progression at 6 mo	1.43	1.19–1.72	**<0.01**	1.69	1.37–2.07	**<0.01**
Progression at 1 y	0.87	0.74–1.01	0.07	0.97	0.82–1.15	0.72
Race White	1.09	0.90–1.32	0.37	1.07	0.85–1.33	0.57
TP53	1.37	1.19–1.58	**<0.01**	1.42	1.20–1.67	**0.02**
KRAS	1.25	1.08–1.47	**<0.01**	1.26	1.05– 1.52	**0.01**
EGFR	0.69	0.58–0.83	**0.03**	0.70	0.58–0.86	**<0.01**
STK11	0.96	0.79–1.18	0.76	1.03	0.81–1.31	0.82
KEAP1	1.10	0.89–1.36	0.36	1.17	0.91–1.49	0.22
LRP1B	0.39	0.22–0.70	**<0.01**	0.35	0.19–0.67	**<0.01**
PRKDC	1.15	0.86–1.54	0.36	1.06	0.73–1.52	0.77
RBM10	1.12	0.83–1.50	0.47	1.15	0.83–1.59	0.41
KMT2D	1.10	0.87–1.38	0.44	0.95	0.72–1.26	0.74

## Data Availability

Data analyzed during the study were provided by a third party. Requests for data should be directed to the provider indicated in the Acknowledgements.

## Abbreviations

AACR	American Association for Cancer Research
CT	Computed Tomography
MR	Magnetic Resonance
NSCLC	Non-Small Cell Lung Cancer
OR	Odds Ratio
OS	Overall Survival
PET/CT	Positron Emission Tomography/Computed Tomography
PSF	Progression Free Survival
